# Projected effectiveness and added value of HIV vaccination campaigns in South Africa: A modeling study

**DOI:** 10.1038/s41598-018-24268-4

**Published:** 2018-04-17

**Authors:** Simon de Montigny, Blythe J. S. Adamson, Benoît R. Mâsse, Louis P. Garrison, James G. Kublin, Peter B. Gilbert, Dobromir T. Dimitrov

**Affiliations:** 1 CHU Sainte-Justine Research Centre, Montreal, Canada; 2 School of Public Health, University of Montreal, Montreal, Canada; 3 The Comparative Health Outcomes, Policy, and Economics (CHOICE) Institute, School of Pharmacy, University of Washington, Seattle, WA USA; 4 Vaccine and Infectious Disease Division, Fred Hutchinson Cancer Research Center, Seattle, USA; 5 Department of Applied Mathematics, University of Washington, Seattle, Washington USA

## Abstract

Promising multi-dose HIV vaccine regimens are being tested in trials in South Africa. We estimated the potential epidemiological and economic impact of HIV vaccine campaigns compared to continuous vaccination, assuming that vaccine efficacy is transient and dependent on immune response. We used a dynamic economic mathematical model of HIV transmission calibrated to 2012 epidemiological data to simulate vaccination with anticipated antiretroviral treatment scale-up in South Africa. We estimate that biennial vaccination with a 70% efficacious vaccine reaching 20% of the sexually active population could prevent 480,000–650,000 HIV infections (13.8–15.3% of all infections) over 10 years. Assuming a launch price of $15 per dose, vaccination was found to be cost-effective, with an incremental cost-effectiveness ratio of $13,746 per quality-adjusted life-year as compared to no vaccination. Increasing vaccination coverage to 50% will prevent more infections but is less likely to achieve cost-effectiveness. Campaign vaccination is consistently more effective and costs less than continuous vaccination across scenarios. Results suggest that a partially effective HIV vaccine will have substantial impact on the HIV epidemic in South Africa and offer good value if priced less than $105 for a five-dose series. Vaccination campaigns every two years may offer greater value for money than continuous vaccination reaching the same coverage level.

## Introduction

The string of successes reported in HIV prevention in the last decade, including the reduction of mother-to-child transmission, interventions like male circumcision, and the introduction of pre-exposure prophylaxis (PrEP), have created optimism that global eradication of HIV is within reach. Nonetheless, many experts argue that adding a vaccine with durable efficacy to the HIV prevention toolbox is essential if the goal is to end the epidemic^1–4^. Investing in global health requires making difficult choices about which initiatives to fund and what level of resources to devote to each initiative^5,6^.

To date, the RV144 Thai trial is the first and only trial to demonstrate partial efficacy of an HIV vaccine. The vaccine efficacy was estimated at approximately 60% in the first year and 31% at the end of follow-up (3.5 years)^7^. The borderline significance of this primary outcome (p = 0.04 for vaccine efficacy differing from zero) posed the question of whether the result could have been a false positive. Standard Bayesian analysis showed that a p-value of 0.04 translates into an approximately 25% chance that the vaccine had no efficacy^8^. However, subsequent analyses, including immune correlates and viral sieve studies, provided additional evidence that the benefit was likely real^9–12^. The confluence of these results would be unlikely if the vaccine efficacy were truly zero; taken together, they support a high likelihood of some beneficial efficacy and a cohesive model of a correlate of protection^13^.

The above studies suggest that modifying the vaccine regimen could improve the vaccine’s level and durability of efficacy. Following the encouraging Thai trial results and the availability of several promising prime-boost HIV vaccine regimens, there is growing interest in designing and implementing randomized efficacy trials comparing one or more vaccine regimens to placebo. A phase 2b/3 randomized controlled trial by the HIV Vaccine Trials Network (HVTN 702) started recruiting participants in October 2016 to evaluate the safety and tolerability of a new vaccine regimen in HIV-seronegative South African men and women and to assess vaccine efficacy (VE) in the first 24 months^14,15^.

The HVTN 702 vaccine regimen was designed to improve the level and durability of protection observed in the Thai trial. It consists of two experimental vaccines: a canarypox vector-based vaccine called ALVAC-HIV and a bivalent subtype C gp120 protein vaccine with MF59 adjuvant to enhance immune response. Both are adaptations of RV144 to the predominant HIV subtype found in southern Africa, namely HIV subtype C. An earlier phase 1 trial (HVTN 100) provided the necessary evidence to launch this large-scale efficacy trial^16^.

Public health authorities are considering various strategies for the rollout of the vaccine. One is a clinic-based continuous vaccination strategy and another is a mass vaccination strategy with periodic campaigns. The campaign being considered in this study would deliver vaccine only for a short period every two years. At manuscript writing, the launch price for an HIV vaccine in South Africa was as yet undetermined.

Mathematical models have been used extensively to study the clinical effectiveness and economic value of biomedical interventions for HIV, such as vaccines, PrEP, and enhanced treatment^17–32^. Cost-effectiveness analyses are used in many fields as economic decision-aid tools to compare the impact of alternative policies and inform decisions on how to maximize returns from limited resources^6,33^. Value frameworks and health technology assessment bodies use cost-effectiveness ratios when evaluating healthcare strategies^34,35^. The incremental cost-effectiveness ratio (ICER) is one such measure. It is calculated as the difference in costs of two alternatives, divided by the difference in their effectiveness. A new intervention is said to be “cost-effective” if the ICER falls below the social willingness-to-pay threshold. The lower the ICER, the better the value. The ICER can also be used when considering a choice of interventions to fund for different diseases. The ICER estimates the average cost per a unit improvement in health, measured in quality-adjusted life-years (QALYs); to determine QALYs, quality of life is assigned a utility weight ranging from 0 to 1, where 1 = optimal health and 0 = a health state equivalent to death^36^.

The objective of the present study was to compare the impact of the two alternative HIV vaccination strategies, for evidence-based decision-making. We first estimated the incidence and prevalence of HIV over the next 10 years in the absence of vaccine. We then compared the effectiveness (HIV infections prevented), efficiency (HIV infections prevented per 1,000 vaccination series) and cost-effectiveness (cost per QALY gained) of a continuous, clinic-based HIV vaccination strategy vs. a mass campaign strategy achieving equivalent vaccination coverage. We extended previous theoretical models of imperfect HIV vaccines^37^ by focusing on concrete implementation strategies and their potential net monetary benefits in real populations. Using simulations, our aim was to outline the conditions whereby vaccination programs could reach various cost-efficiency thresholds.

In this analysis, we used the healthcare payer perspective, namely, the government of South Africa. We included only the costs of incorporating the policy into healthcare coverage but not components such as the cost of vaccine development or patient time. For the willingness-to-pay threshold, as South Africa does not presently have one, we used 1 to 3 times the national gross domestic product (GDP) per capita, as suggested by the World Health Organization (WHO) guidelines^38^.

## Results

### HIV epidemic in the absence of vaccination

We used a deterministic compartmental model to simulate the transmission of HIV in a population of 15–49-year-olds in South Africa, with a system of differential equations representing the flow of individuals between compartments over time. We calibrated our model to the 2012 epidemiological data in South Africa. We assumed current standards of HIV prevention and treatment in South Africa as well as expansion of antiretroviral therapy (ART) eligibility to HIV-infected persons with CD4 T-cell counts below 500.

**Figure 1:**
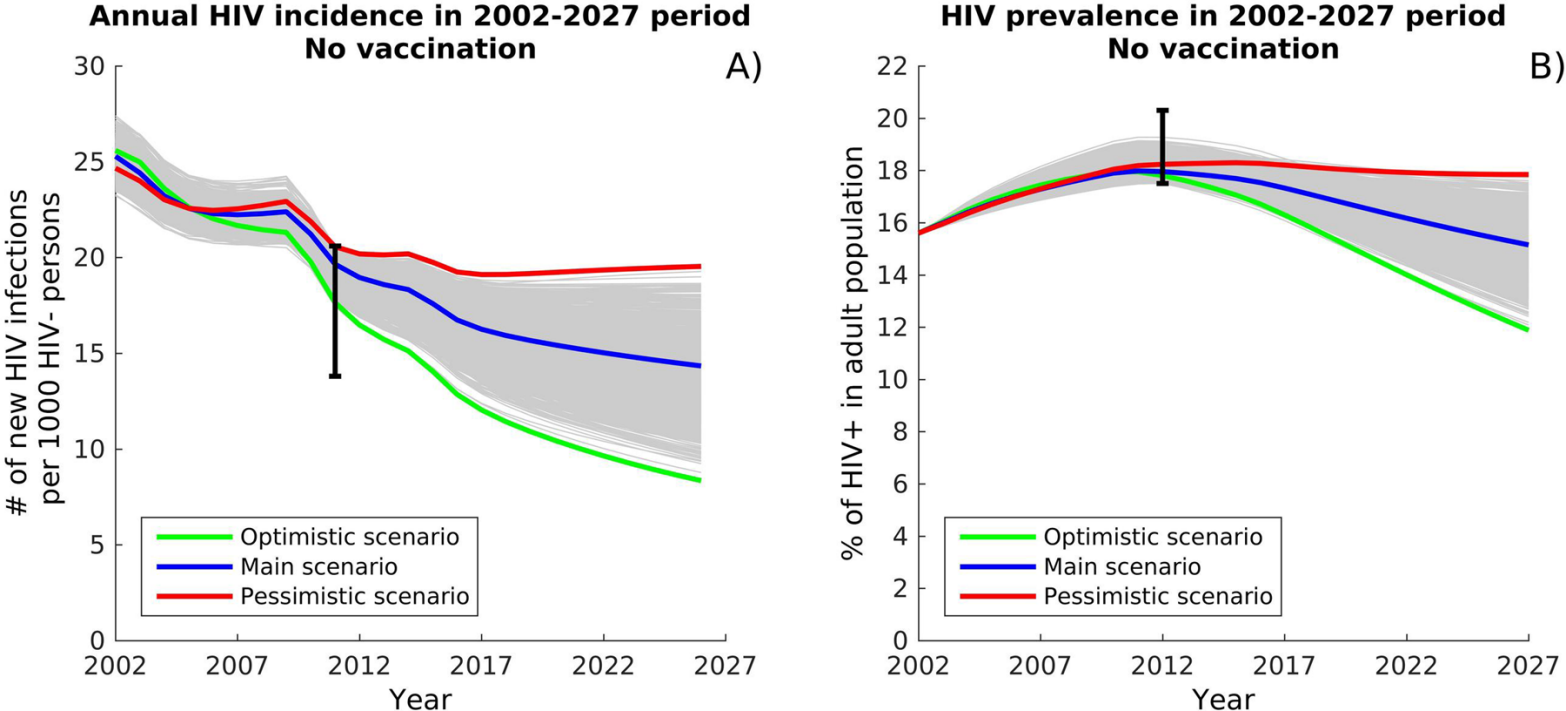
Model calibration. The HIV epidemic in South Africa was projected to 2027 and fitted to 2012 data, showing estimates of: (**A**) HIV incidence, and (**B**) HIV prevalence in the population of 15–49 year-olds, in the absence of vaccine. Three epidemic scenarios (in color) were selected from all 1000 simulations (in gray) and used in the cost-effectiveness analysis: an “optimistic” scenario with declining HIV prevalence and HIV incidence; a “pessimistic” scenario with stable HIV prevalence and slightly rising HIV incidence; and the “main” scenario, represented by the median incidence curve out of all calibrated parameter sets.

To characterize the uncertainty in effectiveness, we projected 1,000 simulations of the HIV epidemic, using various levels of effectiveness of care and prevention. Of these simulations, we selected three for comparison in the cost-effectiveness analysis. One was an “optimistic scenario” assuming higher ART coverage (52.3%) and almost perfect ART efficacy (97.4%). The vast majority of treated individuals would then be virally suppressed, resulting in more effective HIV prevention and a significant decline in HIV incidence and prevalence. Another was a “pessimistic scenario” assuming lower ART coverage (49.4%) and less effective ART (79% efficacy), resulting in less effective HIV prevention and relatively stable or slightly increasing HIV incidence and prevalence. Our “main scenario” was represented by the median incidence curve out of all calibrated parameter sets (Fig. 1). When not otherwise specified, the cost comparisons in this study are based on the main scenario, the median curve.

**Figure 2:**
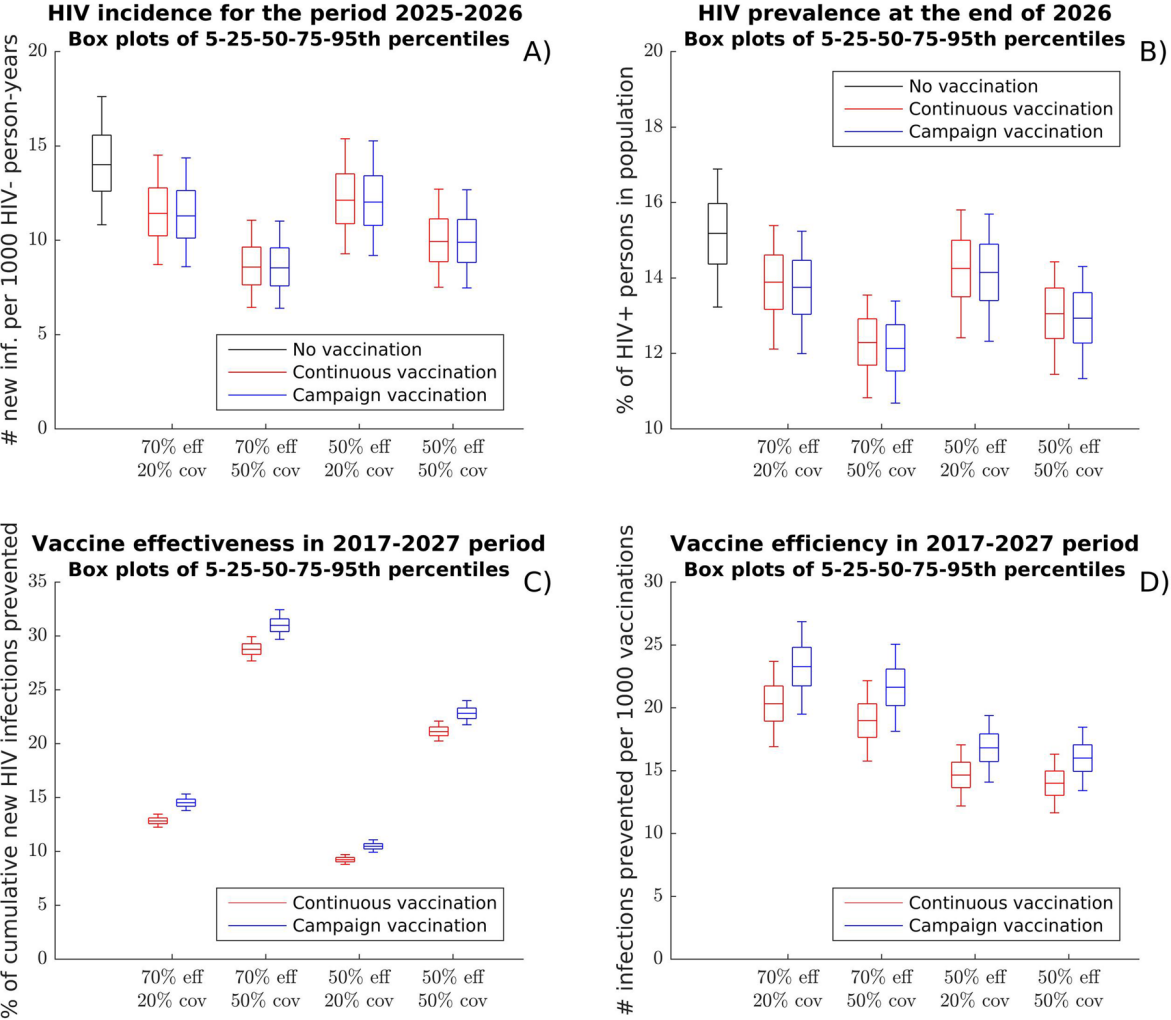
Impact of continuous and campaign vaccination strategies on the HIV epidemic in South Africa. (**A**) HIV incidence after 10 years of vaccination; (**B**) HIV prevalence after 10 years of vaccination; (**C**) Vaccination effectiveness measured as the cumulative fraction of new infections prevented over 10 years; (**D**) Vaccination efficiency measured as the number of infections prevented per 1000 vaccination series over 10 years. Box plots (5th, 25th, 75th, and 95th percentiles) reflect estimated variation over 1000 simulated epidemics. Effectiveness and efficiency for other coverage levels are presented in Supplementary Fig. S5.

As a reference point, or “baseline scenario”, we estimated HIV incidence and prevalence in the next 10 years with no vaccination and standard HIV prevention and care. We obtained an estimated median of 3.9 million new HIV infections occurring between 2017 and 2027 (90% uncertainty interval [UI]: 3.2–4.6). In the year 2026, we would expect a median incidence of 13.9 new HIV infections per 1,000 person-years (90% UI: 10.7–17.6) (Fig. 2A). At the end of the 2017–2027 period, the median HIV prevalence is predicted to be 15.2% of the population (90% UI: 13.2–16.9%) (Fig. 2B).

### Effectiveness and efficiency of HIV vaccination

Figure 2 compares the incidence, prevalence, and projected effectiveness and efficiency of the continuous and campaign vaccination strategies, under scenarios of either low (20%) or high (50%) population coverage and vaccine efficacy profiles averaging low (50% protection) or high (70% protection) VE over two years, as described in Supplementary Fig. S3. All scenarios assume that the vaccine is protective for the 72% of recipients who respond to it, while it has no effect for the 28% of vaccine recipients without an adequate immune response.

Our analysis suggests that in the “base-case scenario” (high VE and low coverage), continuous vaccination will prevent 501,000 infections (90% UI: 425,000–579,000) over a 10-year period. This would result in a modest 18.4% (90% UI: 17.4–19.6%) reduction in HIV incidence over the 2025–2026 period relative to the baseline scenario without vaccination. By comparison, campaign vaccination will prevent 567,000 infections (90% UI: 484,000–650,000) while using 274,000 fewer vaccinations (1.1%) over the same period. Campaign vaccination would reduce HIV incidence for the 2025–2026 period by 19.3% relative to the scenario without vaccination (Fig. 2A).

Alternative vaccination scenarios are considered. Assuming low VE and low coverage decreases the relative reduction in HIV incidence to 13.4% and 14.1% after 10 years of continuous and campaign vaccination, respectively. Assuming low VE and high coverage yields 29.1% and 29.3%, respectively; while high VE and high coverage improves incidence reduction to 38.7% and 39.1%, respectively.

The annual HIV incidence at the end of the 10-year intervention is slightly higher under campaign vaccination, which could be attributed to fluctuations of this metric from year to year as a result of the waning efficacy between vaccination campaigns (Supplementary Fig. S4A). This difference is not informative for the relative impact of each vaccination strategy, as indicated by the comparison of effectiveness and efficiency metrics presented below.

The effectiveness of vaccination measured as the fraction of infections prevented over 10 years would be 12.8% (90% UI: 12.2–13.5%) for continuous vaccination in the base-case scenario (high VE, low coverage), as compared to 14.5% (90% UI: 13.8–15.3%) for campaign vaccination (Fig. 2C). Effectiveness decreases by 27–28% with low VE and low coverage, increases by 112–126% with high VE and high coverage, and increases by 56–65% with low VE and high coverage.

The efficiency of vaccination under the base-case scenario is 20.3 infections prevented/1000 vaccination series for continuous vaccination, as compared to 23.3 for campaign vaccination (Fig. 2D). Efficiency decreases with low VE to 14.6 and 16.0 for continuous and campaign vaccination, respectively.

In all scenarios, campaign vaccination is slightly more effective (in terms of percentage of infections prevented) and more efficient (in terms of infections prevented per 1,000 vaccination series) than continuous vaccination due to instantaneous benefits of the vaccine at the time of the first campaign as compared to a more gradual accumulation of benefits under continuous vaccination. The advantage of campaign vaccination will likely decrease over longer periods of evaluation (see Supplementary Table S2). Modified continuous strategies in which the vaccination rate is increased by up to 50% in the first two years after initiation show improved effectiveness but still remain less efficient than campaign vaccination (Supplementary Table S3).

Notably, the intervention with high VE and high coverage is projected to be 3 times as effective in terms of percentage of infections prevented but only 30% more efficient than the intervention with low VE and low coverage (Fig. 2C,D). On the other hand, whereas the intervention with high VE and low coverage scores considerably behind interventions with high coverage in effectiveness, it is projected to be the most efficient. Additional analysis on campaign vaccination shows that its effectiveness improves with increasing coverage, while its efficiency diminishes somewhat with increasing coverage at high VE while remaining almost flat at low VE (see Supplementary Fig. S5). This suggests that the cost-effectiveness of vaccination programs, which is a reflection of economic efficiency, may not necessarily improve if more people are vaccinated, outlining the need for a formal cost-effectiveness analysis.

### Cost-effectiveness analysis

To estimate the potential cost-effectiveness of HIV vaccines, we added the cost of HIV immunization, testing, and treatment in South Africa to our dynamic transmission model (see Table 1). Benchmarking on the highest-priced routine vaccine in a selection of middle-income countries, we assumed an HIV vaccine price of $15 per dose for the 5-dose series paid for by the government of South Africa^39^. Vaccination costs also include supply chain, service delivery, and rapid HIV testing at each dose.

**Table 1:** Summary of key model parameters.

Parameter	Value	Range	Reference
**I. Epidemic parameters**			
Number of sex acts per year		95–120	^55^
Proportion of sex acts protected by condom	20%		^56^
Condom efficacy	70%		^57^
Infection probability per act by HIV stage and CD4 count:			Estimated^58^; see also Supplementary Information
Acute stage	5.5%		
Recent infections and asymptomatic stage (CD4 > 500)	0.21%		
Asymptomatic stage (CD4 350–500)	0.06%		
Asymptomatic stage (CD4 200–350)	0.11%		
Symptomatic stage (CD4 < 200)	0.33%		
Efficacy of antiretroviral therapy (ART) in reducing infectiousness		73–99%	^59^
Vaccine coverage	20%	20% or 50%	Assumed
Average vaccine efficacy (VE) in reducing susceptibility over a 2-year period	70%	50% or 70%	Assumed^7,15^
Proportion of vaccinated population responding to the vaccine	72%		HVTN 702 protocol
**II. Economic parameters**			
Costs per programmed dose, total^*a*^	$30		
Vaccine price	$15	$1–$30	Assumed with reference pricing^39^
Supply chain	$0.60		^39^
Service delivery^*b*^	$5.07	$1–$10	^60^
HIV test, facility-based	$9.30	$5–$15	^61,62^
Cost of HIV treatment per year, total	$718	$648–$789	^54^
ART	$191	$172–$210	^54^
Personnel	$352	$317–$387	^54^
Labs	$107	$97–$118	^54^
Other	$68	$62–$75	^54^
Utility weights (health state)			Estimated^49^
Acute HIV	0.800	0.702–0.935	
CD4 count > 350	0.935	0.821–1.0	
CD4 count 200–349	0.818	0.723–0.912	
CD4 count < 200	0.702	0.567–0.837	
Discount rate	3%	0–5%	^44,52^

^a^Costs adjusted to 2017 USD.

^b^Costs of delivery per vaccine dose include needle, syringe and alcohol swab for administration.

When we considered the costs and benefits of vaccination over a 10-year period (Table 2), the campaign strategy consistently dominated continuous clinic-based delivery providing greater health benefits at lower costs than continuous vaccination, and this held true for all scenarios. In the base-case scenario (high VE, low coverage), a biennial HIV vaccine campaign could cost the healthcare payer $14.7 billion over 10 years. Standard care for HIV with no vaccine would cost $11.7 billion. Vaccination would therefore cost an additional $117 per eligible adult. It would add 223,624 QALYs in the population, or 0.0085 QALYs per eligible adult, avoiding over 44,000 deaths due to AIDS.

**Table 2:** Cost-effectiveness results.

OUTCOME	BASE-CASE SCENARIO			ALTERNATIVE SCENARIOS					
	No Vaccine	70% VE and 20% Coverage		50% VE and 20% Coverage		50% VE and 50% Coverage		70% VE and 50% Coverage	
		Clinic-Based Vaccination	Campaign Vaccination	Clinic-Based Vaccination	Campaign Vaccination	Clinic-Based Vaccination	Campaign Vaccination	Clinic-Based Vaccination	Campaign Vaccination
Vaccinated adults (millions)	—	24.72	24.45	24.68	24.40	59.17	55.92	59.40	56.13
Total cost (billions $)	$11.7	$14.7	$14.7	$14.8	$14.8	$19.2	$18.8	$19.1	$18.7
Total QALYs (millions)	269.3	269.4	269.5	269.4	269.4	269.6	269.6	269.7	269.8
AIDS deaths (millions)	2.86	2.82	2.81	2.83	2.83	2.80	2.78	2.78	2.76
Incremental cost (billions $)	—	$3.1	$3.1	$3.1	$3.1	$7.5	$7.2	$7.4	$7.1
Per person vaccinated ($)		$124.99	$125.74	$126.56	$127.90	$126.92	$128.25	$125.40	$126.21
Per eligible adult ($)^a^		$117.20	$116.58	$118.46	$118.38	$284.82	$272.03	$282.49	$268.69
Incremental QALYs, total	—	165,856	223,624	117,988	159,423	285,325	361,398	394,889	498,889
Per person vaccinated		0.0067	0.0091	0.0048	0.0065	0.0048	0.0065	0.0066	0.0089
Per eligible adult^a^		0.0063	0.0085	0.0045	0.0060	0.0108	0.0137	0.0150	0.0189
AIDS deaths avoided	—	32,388	44,480	23,001	31,678	56,195	72,183	77,968	99,797
ICER ($/QALY)		Dominated^b^	$13,746	Dominated^b^	$19,578	Dominated^b^	$19,846	Dominated^b^	$14,200

Assumptions: vaccine price, $15 per dose; median test-and-treat impact; $ in 2017 USD.

Abbreviations: QALY, quality-adjusted life-year; ICER, incremental cost-effectiveness ratio.

^a^Eligible adults: In this table, eligible adults refers to the total number of adults age 15–49 in South Africa, regardless of coverage level.

^b^Dominated: Of two strategies, the “dominated” strategy is not clinically superior and has higher costs than the comparison strategy.

**Figure 3:**
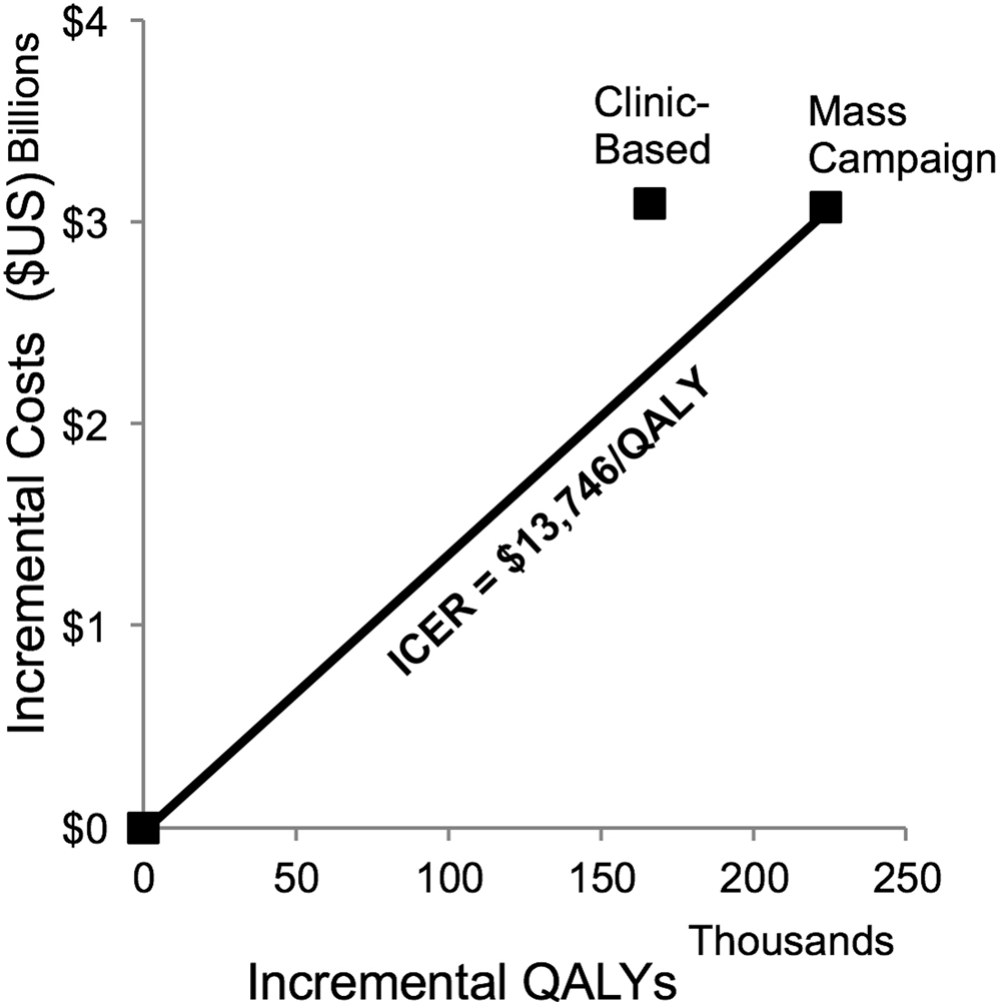
Cost-effectiveness of campaign vaccination versus continuous clinic-based HIV vaccine delivery. The mass campaign vaccination dominated continuous clinic-based delivery and was cost-effective using a threshold of 3 × gross domestic product (GDP) per capita. ICER, incremental cost-effectiveness ratio; QALY, quality-adjusted life-year.

A willingness-to-pay threshold of 3 × GDP/capita is equivalent to $16,767/QALY; a threshold of 1 × GDP/capita works out to $5,589/QALY. In the base-case scenario, the incremental cost-effectiveness ratio (ICER) for an HIV campaign-based vaccine was $13,746/QALY, as compared to standard of care with no vaccination (Table 2; Fig. 3). This is cost-effective on a 3 × GDP/capita threshold, but not on a 1 × GDP/capita threshold. The vaccination is unlikely to be cost-effective in the optimistic epidemic scenario ($18,948/QALY) but is potentially more cost-effective in the pessimistic epidemic scenario ($10,925/QALY), depending on the willingness-to-pay threshold used.

A low efficacy vaccine is unlikely to be cost-effective if it costs $15 per dose in epidemic settings where HIV treatment and prevention are successful (optimistic epidemic scenario). Simulations of HIV vaccine campaigns with low efficacy and low coverage yield an ICER of $19,578 per QALY, as compared to standard care with no vaccine (Table 2).

Expanding population coverage achieves substantial benefits to the population but also requires additional healthcare resources. As a result, scenarios with 20% and 50% coverage produced similar cost-effectiveness outcomes (Table 2; Supplementary Fig. S6). Although vaccination reduces the 10-year health sector expenditures on ART due to fewer HIV infections, the amount saved is less than the added cost of vaccinating an additional 30% of the population (Supplementary Fig. S6).

### The value of campaign vaccination

The service delivery costs for HIV campaign and continuous clinic vaccination may not actually be the same, as assumed above, and the potential difference in cost is uncertain. To inform the choice of implementation strategies, we quantified the added value of a mass campaign, as compared to continuous clinic-based delivery, using a net monetary benefit (NMB) approach. If decision-makers were to determine that the additional cost of a campaign exceeded the added benefit, then clinic-based implementation would be the better choice. Using a willingness-to-pay threshold of 3 × GDP/capita, we calculated the NMB of the campaign strategy as monetized health benefits (QALYs gained multiplied by the willingness-to-pay threshold) less the incremental costs as compared to the continuous strategy. If service delivery costs were the same for both strategies, the NMB of a campaign strategy would be $985 million, or $37 per eligible adult, as compared to a continuous strategy. Lower NMB values are expected with low efficacy and low coverage scenarios and higher ones with high efficacy and high coverage scenarios ($697 million and $2.1 billion, respectively).

### Sensitivity analysis

**Figure 4:**
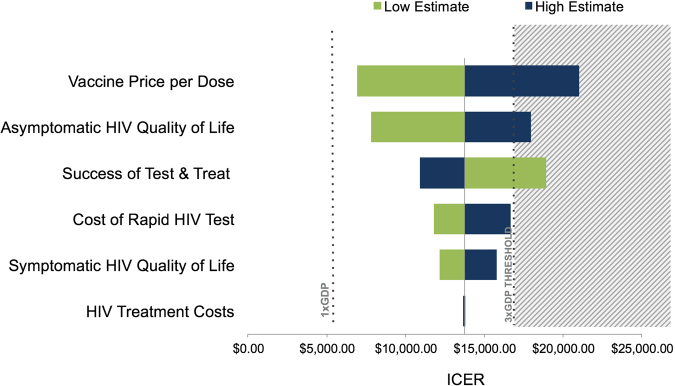
HIV vaccine cost-effectiveness: Tornado plot of univariate sensitivity analysis. Incremental cost-effectiveness ratios (ICERs) for HIV vaccinations were calculated with 2 values for each parameter: the lowest in the range (green) and highest in the range (dark blue) while the rest of the parameters were fixed at their base-case (70% vaccine efficacy, 20% coverage) values. The mean base-case ICER is represented by the solid vertical line. The region with the grey background represents vaccinations that are not cost-effective (using a 3 × GDP/capita threshold).

We used a univariate sensitivity analysis to assess the effect of individual parameter uncertainty on estimated costs, QALYs, and ICER for each strategy. We found that the main driver of cost-effectiveness was vaccine price. The value of an HIV vaccination (as measured by the ICER) was also sensitive to the quality of life with asymptomatic HIV, and to the success of test and treat programs (Fig. 4). ICERs in various scenario analyses (Supplementary Table S4) ranged from $9,183 to $36,882 per QALY. Increasing the coverage from 20% to 50% increased the total cost but did not substantially affect cost-effectiveness (Table 2). To estimate the maximum vaccine price for the vaccination to remain cost-effective, we varied price while holding all other parameters fixed. The threshold price was $21/dose, ranging from $11/dose in our pessimistic scenario to $29/dose in our optimistic ART scale-up scenario. Without reductions in the costs of service delivery or HIV testing, the vaccine purchase price would need to be less than $1 per dose to be cost-effective using a 1 × GDP/capita threshold (Fig. 4). A probabilistic sensitivity analysis that generated 1000 Monte Carlo simulations varying costs and utilities of 24 scenarios confirmed that vaccination in low-incidence settings was less likely to be cost-effective than in high-incidence settings (Supplementary Fig. S7). There were no scenarios in which HIV vaccines were cost-saving.

## Discussion

Epidemic projections based on currently available HIV treatment and prevention methods suggest that HIV elimination is highly unlikely without the development and introduction of an effective preventive vaccine^1,2^. However, healthcare systems have a limited pool of funding resources available for HIV prevention and treatment. New innovations must therefore show not only effectiveness but also value for money spent. Using economic models as decision-aid tools, policymakers and healthcare payers can optimize HIV care and prevention portfolios while maximizing long-term return on investment. Recent intervention programs focussing on universal access to treatment and increased delivery of ART to HIV-positive individuals have to be balanced with effective HIV prevention strategies. In this study, we used a cost-effectiveness approach to assess the potential impact of two HIV vaccination strategies, assuming various levels of vaccine efficacy, population coverage, and projections of existing HIV prevention and care. We based our model on a vaccine currently being tested in clinical trials in South Africa. Our analysis shows that campaigns rolled out every two years would likely be less costly and provide greater health benefits than continuous on-demand vaccinations reaching the same coverage. Results suggest that even a partially effective HIV vaccine will have substantial impact on the HIV epidemic in South Africa and will offer good value in the base-case scenario if priced less than $105 per five-dose series. It could potentially avoid over 44,000 deaths from HIV in South Africa over the next 10 years.

There are several conditions under which HIV vaccination programs could be cost-effective. We projected that vaccinating 20% of the sexually active population with a 70% efficacious vaccine would prevent approximately 500,000 infections over the next 10 years, representing 14% of the expected new HIV infections in South Africa. Not surprisingly, the effectiveness of the intervention, measured as the proportion of infections prevented, increased for higher vaccine efficacy and rollout coverage. However, the analysis of the average intervention efficiency, measured as the number of infections prevented per 1000 vaccination series, tells a different story. Efficiency still improves with better vaccine efficacy because more infections are prevented with the same number of vaccinations. However, efficiency does not necessarily improve with higher coverage because in this case, more infections are prevented due to more vaccination series being distributed. In fact, our analysis projects that vaccine efficiency would decline with improved vaccination coverage.

We performed a cost-effectiveness analysis to see which vaccination strategies provided better health outcomes in a setting with limited allocation resources. The results showed that the population-level net monetary benefit from HIV vaccination was sensitive to the timing of the implementation. It also showed that biennial mass campaign vaccination in South Africa was likely to be cost-effective if vaccine efficacy was at least 70% and the vaccine priced less than $21 per dose, or totalling less than $105 per series. The analysis indicated that vaccination campaigns every two years may offer greater value for money than continuous vaccination reaching the same coverage level. Not surprisingly, interventions with a less effective vaccine would be less cost-effective. Notably, increasing coverage from 20% to 50% of the population would prevent more HIV infections but would also decrease the likelihood of cost-effectiveness, especially if currently available prevention and treatment programs were projected to reduce HIV incidence in the absence of a vaccine. Our analysis outlines the importance of the epidemic context when a vaccine is introduced. We believe that expected delays in vaccine availability will only increase the discrepancies between the optimistic and pessimistic epidemic scenarios, hence the urgency for rapid development and deployment of HIV vaccines^25^.

A recent cost-effectiveness analysis evaluated a similar HIV vaccine regimen targeting adolescents in South Africa^29^. Our results suggest that vaccination is less likely to be cost-effective in our model than in theirs. The disparity is likely attributable to differences in the assumed durability of protection (2 years in ours vs. 10 years in theirs) and in time horizon (10 years in ours vs. lifetime in theirs) between models.

This study has some limitations that may affect projected effectiveness and cost-effectiveness. First, our model was not stratified by gender, age, or high-risk groups to target, so for specific, targeted HIV “low coverage” vaccinations, actual costs and benefits may fluctuate from those presented. However, our analysis suggests that the differences between vaccine delivery (continuous and campaign) as well as between the various scenarios (VE, coverage, projections) result from an assumed distribution of vaccinations over time. These assumptions are unlikely to be affected by age or gender distributions. Second, the cost of an HIV vaccine was a major contributor to the uncertainty in the cost-benefit projections. It is possible that non-linear recurrent costs could occur at different levels of coverage of either vaccination strategy. Experience from the introduction and scaling-up of human papillovirus (HPV) vaccines suggests that a wide range of recurrent costs could occur per immunized individual due to differences in scope and scale, strategy, national income, and health system policies and program^40^. This and other limitations in data will be informed after a vaccine becomes available. Model results should be read as approximated projections of the total costs for each strategy aiming to inform stakeholders in the vaccine development process about the monetized benefits of herd immunity provided by an aggressive campaign vaccination. Third, in other countries, the cost components and willingness-to-pay thresholds may be different from those in South Africa. The cost-effectiveness results presented here are therefore not easily transferable to other settings. Further, vaccine pricing assumptions were based on expert opinion benchmarked in reference to the average pneumococcal conjugate vaccine price in middle-income countries. To account for this uncertainty, we included a wide range of costs in the sensitivity analysis. The model does not include HIV-unrelated health care costs, underestimating the total healthcare costs of HIV-infected individuals and likely underestimating the incremental cost offsets from infections prevented. Finally, we did not conduct a budget impact analysis for South Africa as it was beyond the scope of this study. As a result, we cannot draw conclusions about individual or health system affordability of HIV vaccines.

Our analysis is a systematic attempt to understand the potential population benefits of the multi-dose HIV vaccine regimen currently being tested in randomized controlled efficacy trials in South Africa. Despite the uncertainty in various epidemic and costing parameters, we demonstrated that a partially efficacious vaccine which requires periodic boosting would have a substantial impact on the HIV epidemic in South Africa, and we provided estimates of the vaccine costs to support decision-making for economically efficient implementation strategies. We would like to emphasize that the impact of future vaccination must be considered only in combination with other HIV prevention methods and, more specifically, with PrEP. Further investigations into more comprehensive HIV prevention strategies will be warranted with the expansion of the HIV-prevention toolbox. Strategies considered for implementation, however, must be based not only on cost-effectiveness but also on plausible levels of acceptability and retention. For instance, oral PrEP has demonstrated problems with acceptance and adherence in Sub-Saharan Africa, particularly among young women. The HIV vaccine has the potential to resolve some of those issues when it becomes available. Economic evaluation of the combined use of PrEP and HIV vaccines will have to take into account the complexity of the problem and more specifically, the hypothetical nature of efficacy estimates and attrition rates for PrEP, long-term vaccine boosting, and the combination of PrEP with vaccines^41^. By defining conditions for cost-effective vaccinations, this study can be used to inform public-health policy on allocating HIV prevention resources. We believe that these questions will receive more attention with forthcoming HIV vaccine development and availability and with time, as the vaccine’s mode of action becomes more clearly defined.

## Methods

This article conforms to the Consolidated Health Economic Evaluation Reporting Standards (CHEERS) Statement of the International Society of Pharmacoeconomics and Outcomes Research (ISPOR)^42^, The analysis was done according to Dynamic Transmission Modeling as outlined in the Report of the ISPOR-SMDM Modeling Good Research Practices Task Force-5^43^. The epidemic model was programmed in MATLAB R2015b (MathWorks Inc., Massachusetts, USA) with economic analyses conducted using Excel version 15.39 (Microsoft®, Redmond, USA) and R version 3.4.2 (R Foundation for Statistical Computing). The values, ranges, and references for key model parameters are reported in Table 1 and Supplementary Table S1.

### Study population, timeframe, and perspective

We examined the impact of a vaccine intervention rolled out to sexually active 15–49-year-olds in South Africa. In accordance with WHO guidelines for cost-effectiveness analyses, the study timeframe was 10 years, i.e. the period from 2017 to 2027^44^. We considered costs from a healthcare payer perspective, i.e. the government of South Africa.

### Dynamic model

We developed a deterministic compartmental mathematical model of HIV transmission in a population of sexually active individuals in South Africa (Supplementary Fig. S2). The model stratified individuals according to HIV infection status and progression towards AIDS. Infected individuals were stratified by treatment status: undiagnosed, diagnosed but not on treatment, diagnosed on treatment, and diagnosed who failed treatment. Untreated individuals (undiagnosed, diagnosed, or diagnosed who failed treatment) were divided into CD4 groups by their actual CD4 count. Treated individuals were assigned the CD4 classification to which they would return if treatment was interrupted. Uninfected individuals were classified by vaccination response (responders or non-responders) and vaccination status (vaccinated or not). Vaccinated individuals go through three successive phases with differential vaccine efficacy before returning to the unvaccinated compartment.

Transmission of HIV was modeled under the assumption that each unprotected sex act between an uninfected and infected partner carries the same risk of infection. The rate of HIV transmission is dependent on the annual rate of new partner acquisition, the average number of sex acts per partnership, the protection conferred by the vaccine (if vaccinated), the fraction of sex acts protected by condoms and the HIV prevalence between partners. Other HIV prevention options such as PrEP and male circumcision were not modeled explicitly. The effects of ART on HIV transmission were modeled by reducing the outgoing infection probability by a fixed percentage (ART efficacy) and substantially increasing the life expectancy of people on HIV treatment. Individuals who interrupted or failed treatment were returned to the usual disease progression process.

Vaccine protection was based on vaccine efficacy data from the RV144 trial. We assumed that response to vaccination was a predetermined characteristic; an individual either does or does not respond, and responds in the same way to the first or subsequent doses.

### Parameters and calibration

We calibrated our dynamic model using the most recent epidemiological data in South Africa, the 2012 South African National HIV Prevalence, Incidence and Behaviour Survey^45^. We estimated the size of the South African adult population using data from the 2015 World Bank^46^. We also used the most recently available data for parameters such as HIV prevention (e.g. condom use), frequency of sex acts, and ART initiation/dropout/failure rates. In the epidemic simulations (starting in 2002), we assumed that ART was initially offered only to infected individuals with CD4 < 200 cells per mm^3^, with eligibility expanded to individuals with CD4 < 350 in 2010 and CD4 < 500 in 2015. The uncertainty in future projections of ART coverage was investigated in a sensitivity analysis by exploring scenarios with different background HIV incidence in the absence of vaccination.

Monte Carlo filtering was used to select 1000 parameter sets closely matching the 2012 epidemiological data (see Supplementary Information). For the purposes of exploring cost-effectiveness for the range of plausible settings in the absence of vaccination, we selected three of the 1000 parameter sets: an “optimistic scenario” with declining HIV prevalence and HIV incidence; a “pessimistic scenario” with stable HIV prevalence and slightly rising HIV incidence; and the “main scenario” represented by the median incidence curve out of all calibrated parameter sets (Supplementary Information and Fig. 1).

### Vaccine protection and implementation strategies

Using immune response targets from the design of the ongoing HVTN 702 trial, we assumed a partial protection in 72% of vaccinated individuals, while the remaining 28% have no response^47^. We simulated time-dependent vaccine efficacy profiles in responders to account for multi-dose scheduling. In the base-case scenario, we achieved an average 70% VE over 2 years, assuming 45% HIV risk reduction over the first 6 months, 95% HIV risk reduction over the next 12 months, and 45% HIV risk reduction over the last 6 months (Supplementary Fig. S3). We also considered a less effective scenario with 50% average VE, assuming 20% HIV risk reduction over the first 6 months, 80% HIV risk reduction over the next 12 months, and 20% HIV risk reduction over the last 6 months. Conservatively, no VE is assumed after the 2-year period. The VE parameter is the multiplicative reduction in the annual per-partnership transmission rate (vaccinated vs. not vaccinated uninfected partner in a serodiscordant partnership) following a leaky mechanism of efficacy^48^.

We investigated two strategies of vaccine deployment: 1) availability in clinics on demand, implying continuous vaccination at a certain rate; and 2) distribution by mass vaccination campaigns that occur every two years. For each deployment scenario, we simulated two vaccine coverage levels: 20% and 50% of the population. Low coverage may represent targeted vaccination strategies, while high coverage represents a vaccine offered to the entire sexually active population. In the base-case scenario, we assumed 20% vaccine coverage and 70% average VE.

### Effectiveness, efficiency and health outcome metrics

Each vaccination strategy was compared to the reference scenario that assumed no vaccination and no changes in current South African guidelines for HIV prevention and treatment (with ART eligibility for CD4 < 500 cells per mm^3^ maintained until 2027). Vaccination effectiveness was measured as the cumulative number and fraction of HIV infections prevented by the vaccination. Vaccine efficiency was measured as number of infections prevented per 1000 vaccination series. All metrics were compared across different scenarios of VE and coverage; results were reported as the median and 90% uncertainty interval (UI, 5^th^–95^th^ percentile) of all 1000 simulations using preselected sets of epidemic parameters identified in the calibration procedure.

To capture improved survival and quality of life in a measure comparable to other diseases, we estimated population-level QALYs calculated by multiplying CD4 count strata-specific preference-based utilities by the compartment size in each health state at quarterly time steps (Table 1)^49^. We assumed no reduction in quality of life from HIV vaccination because clinical trials have shown minimal side effects^7,15^. For the 10-year time horizon of 2017–2027, potential costs and QALYs for each scenario were discounted 3% annually to reflect the net present value^44,50,51^. As the dynamic transmission model is not stratified by age, all susceptible individuals were assigned one health state utility weight, regardless of age. QALYs were compared across main, optimistic and pessimistic epidemic scenarios with different VE and coverage.

### Costs

Costs were projected from a healthcare payer perspective, i.e. the South African government, because it will be purchasing the product, paying for immunization, deciding the targeting policy, and defining the strategy for implementation. In the past, this viewpoint was called a “payer perspective.” Recently, best practice guidelines have changed the standard and now call this viewpoint a “healthcare sector perspective”^51^. Costs are in USD, with 2017 as the base year. Future costs were discounted at 3% each year to reflect present value, in accordance with WHO guidelines^44,51,52^. Unit costs for doses administered include the vaccine price, supply chain, and service delivery (Table 1)^39^. The launch price of an HIV vaccine for South Africa is uncertain. We assumed an average wholesale price per dose as $15 in the base case by benchmarking the price of the pneumococcal conjugate vaccine, the highest price routine vaccine, in non-GAVI eligible middle-income countries. Price sensitivity was examined in univariate and multivariate sensitivity analyses^53^. Supply chain costs were set at $0.60 per dose^39^. For the continuous immunization strategy, clinic-based service delivery for adults includes needle, syringe and alcohol swab for administration, averaging $5.07 per dose. We assumed that a rapid HIV diagnostic test would be required before each dose. HIV-associated healthcare expenditures were based on South African costing data from the MATCH study^54^. HIV costs include drugs, personnel time, labs, and other health expenditures across all stages of disease (Table 1)^54^. The cost of vaccine administration per dose was assumed to be the same in continuous and campaign implementation strategies.

### Cost-effectiveness analysis

The incremental cost-effectiveness ratio (ICER) is defined here as the incremental cost divided by the incremental benefit in QALYs, comparing each strategy to the option with the next best health outcome. ICER is a measure of economic efficiency (value for money spent).

1 $$ICER=\frac{Cos{t}_{intervention}-Cos{t}_{standardofcare}}{QAL{Y}_{intervention}-QAL{Y}_{standardofcare}}.$$

South Africa has not explicitly defined a willingness to pay for health, and we used a range of 1–3 times the GDP per capita of South Africa ($5,589) for cost-effectiveness interpretation^44,50^.

We used ICERs to compare a given vaccine strategy to the next best option. For example, the ICER for the scenario with 70% effective vaccine and 50% coverage describes the marginal costs and QALYs as compared to the scenario with 70% effective vaccine and 20% coverage.

The net monetary benefit (NMB) for a vaccine campaign compared to continuous vaccination is defined by the following equation, where WTP is the willingness-to-pay threshold:

2 $${\rm{NMB}}\,=({{\rm{QALYs}}}_{{\rm{campaign}}}-{{\rm{QALYs}}}_{{\rm{continuous}}})\,\ast \,{\rm{WTP}}\,\mbox{--}\,({{\rm{Cost}}}_{{\rm{campaign}}}-{{\rm{Cost}}}_{{\rm{continuous}}}).$$

To monetize the value of campaign vaccination over continuous vaccination per dose delivered, we conducted a threshold analysis, varying the cost of vaccine administration to find the maximum value that would remain cost-effective. This benefit is the economic equivalent of a positive externality.

### Sensitivity analysis

To explore the impact of uncertainty on cost-effectiveness, we used a univariate sensitivity analysis varying key parameters individually from the lowest to highest values of their range (Table 1). The effects on incremental costs, QALYs, and ICERs were sorted from most to least influential and graphed as a tornado plot (Fig. 4). To incorporate uncertainty in efficacy, coverage, delivery strategy, and HIV incidence, the univariate sensitivity analysis was repeated with various scenarios of each combination. A threshold analysis was performed to estimate the maximal vaccine price at which a vaccination program would be cost-effective. In this analysis, the price per HIV vaccine dose was raised until the intervention ICER was equal to 3 × GDP/capita of South Africa. We further evaluated combined parameter uncertainty for different combinations of VE (50% and 70%), vaccination coverage (20% and 50%), implementation strategy (campaign and continuous), and epidemic setting (main, optimistic, pessimistic).

## Electronic supplementary material

Supplementary Information
