# Supplementary material for: Projected effectiveness and added value of HIV vaccination campaigns in South Africa: A modeling study
Source: Sci Rep. 2018 Apr 17;8:6066. doi: 10.1038/s41598-018-24268-4 (PMC5904131; doi:10.1038/s41598-018-24268-4)
Supplement: Supplementary file 1 — Supplementary Information [file 41598_2018_24268_MOESM1_ESM.pdf]

Supplementary Information

Projected effectiveness and added value of HIV vaccination campaigns in South Africa: A modeling study

Simon de Montigny, Blythe J.S. Adamson, Benoît R. Mâsse, Louis P. Garrison, Jr, James G. Kublin, Peter B. Gilbert, Dobromir T. Dimitrov

1. Comparison of vaccination delivery schedules

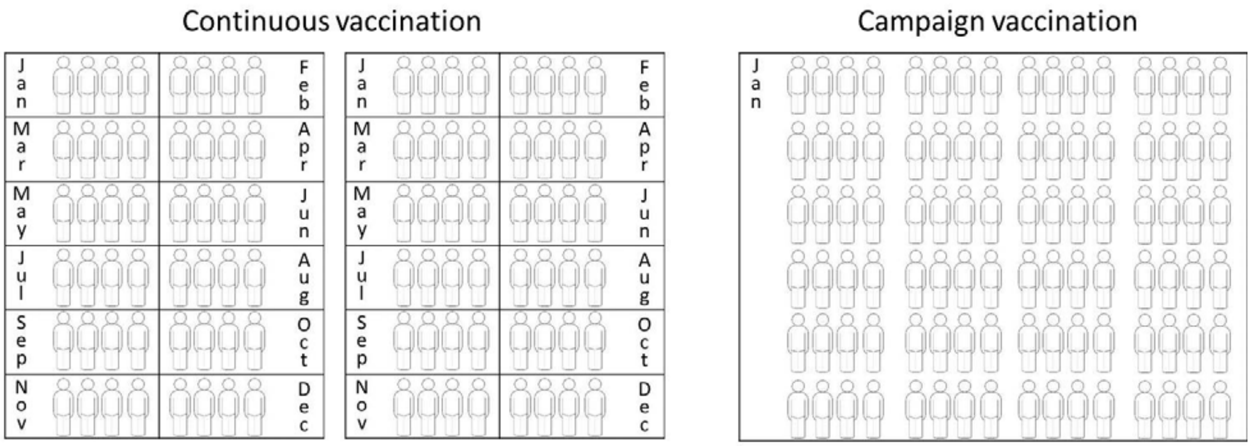

Figure S1 Diagram illustrating difference between vaccination delivery schedules. In the clinic-based vaccination delivery (left panel) the vaccine is available on demand, such that individuals are continuously vaccinated at a certain rate. In the simulation of campaign vaccination (right panel), the individuals are moved from susceptible compartment to the first vaccinated compartment (in both immune response groups) instantly at the beginning of the 2-year period to approximate in mass campaigns over a short time period.

2. Epidemic model description

Model Diagram

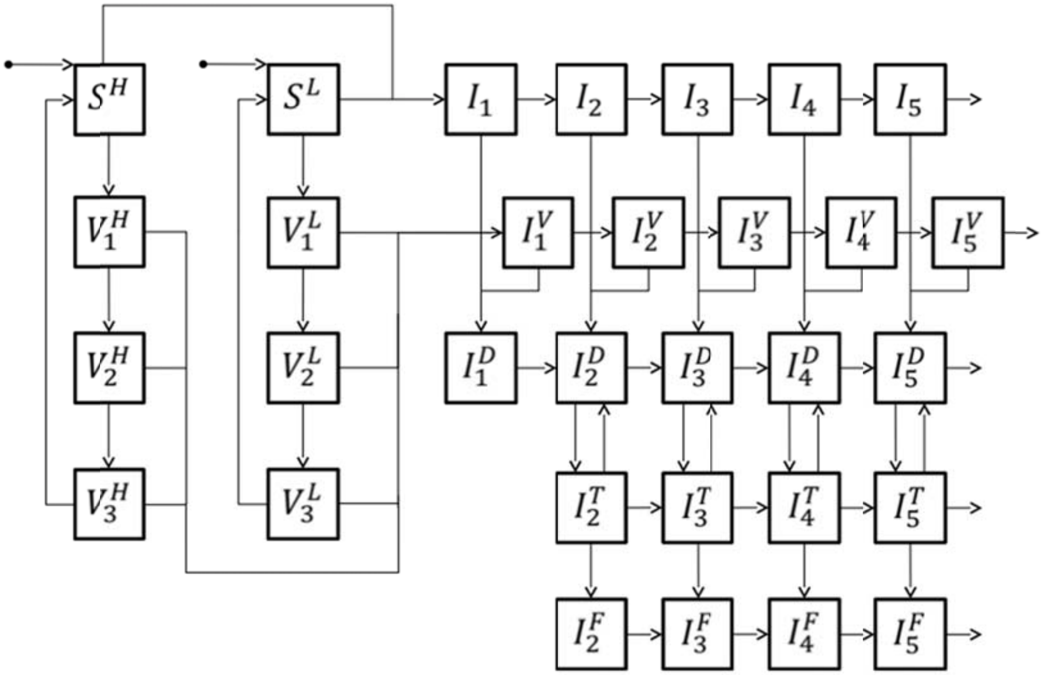

Figure S2 Flow diagram of the model. Simulated population is stratified in compartments by vaccine and HIV status as susceptible unvaccinated (S), susceptible vaccinated (V) and infected (I). Vaccinated individuals are divided by their immune response to the vaccine in high (superscript H) and low (superscript L) response groups. Each vaccinated individual goes through 3 stages of vaccine protection (subscripts 1-3) with different level of VE determined by the VE profile (Fig. S2). Infected individuals progress through 5 stages of infection (subscripts 1-5) determined by level of CD4 count (acute, CD4 >500, CD4 350-500, CD4 200-350, CD4 <200). Infected individuals are additionally stratified as infected while vaccinated (superscript V), diagnosed (superscript D). on ART (superscript T) and failed ART (superscript F). A complete description of the model including the expressions for the forces of infections (λ) is presented below.

Model equations

$$\frac{d}{dt}S^H = f_H\pi N + \tau_3^H V_3^H - (\lambda_0^H + \tau_0^H + \mu)S^H$$
$$\frac{d}{dt}V_1^H = \tau_0^H S^H - (\lambda_1^H + \tau_1^H + \mu)V_1^H$$
$$\frac{d}{dt}V_2^H = \tau_1^H V_1^H - (\lambda_2^H + \tau_2^H + \mu)V_2^H$$
$$\frac{d}{dt}V_3^H = \tau_2^H V_2^H - (\lambda_3^H + \tau_3^H + \mu)V_3^H$$
$$\frac{d}{dt}S^L = (1 - f_H)\pi N + \tau_3^L V_3^L - (\lambda_0^L + \tau_0^L + \mu)S^L$$
$$\frac{d}{dt}V_1^L = \tau_0^L S^L - (\lambda_1^L + \tau_1^L + \mu)V_1^L$$
$$\frac{d}{dt}V_2^L = \tau_1^L V_1^L - (\lambda_2^L + \tau_2^L + \mu)V_2^L$$
$$\frac{d}{dt}V_3^L = \tau_2^L V_2^L - (\lambda_3^L + \tau_3^L + \mu)V_3^L$$
$$\frac{d}{dt}I_1 = \lambda_0^H S^H + \lambda_0^L S^L - (\nu_1 + \delta_1 + \mu)I_1$$
$$\frac{d}{dt}I_k = \nu_{k-1}I_{k-1} - (\nu_k + \delta_k + \mu)I_k\ ;\ k = 2,3,4,5$$
$$\frac{d}{dt}I_1^V = \sum_{\substack{k=1,2,3\\ E=H,L}} \lambda_k^E V_k^E - (\nu_1^V + \delta_1^V + \mu)I_1^V$$
$$\frac{d}{dt}I_k^V = \nu_{k-1}^V I_{k-1}^V - (\nu_k^V + \delta_k^V + \mu)I_k^V\ ;\ k = 2,3,4,5$$
$$\frac{d}{dt}I_1^D = \delta_1 I_1 + \delta_1^V I_1^V - (\nu_1^D + \mu)I_1^D$$
$$\frac{d}{dt}I_k^D = \nu_{k-1}^D I_{k-1}^D + \delta_k I_k + \delta_k^V I_k^V + \delta_k^T I_k^T - (\nu_k^D + \gamma_k^D + \mu)I_k^D\ ;\ k = 2,3,4,5$$
$$\frac{d}{dt}I_2^T = \gamma_2^D I_2^D - (\delta_2^T + \nu_2^T + \theta_2^T + \mu)I_2^T$$
$$\frac{d}{dt}I_k^T = \nu_{k-1}^T I_{k-1}^T + \gamma_k^D I_k^D - (\delta_k^T + \nu_k^T + \theta_k^T + \mu)I_k^T\ ;\ k = 3,4,5$$
$$\frac{d}{dt}I_2^F = \theta_k^T I_k^T - (\nu_k^F + \mu)I_k^T$$
$$\frac{d}{dt}I_k^F = \nu_{k-1}^F I_{k-1}^F + \theta_k^T I_k^T - (\nu_k^F + \mu)I_k^T\ ;\ k = 3,4,5$$
$$N = \sum_{k=0}^{3} V_k^H + \sum_{k=0}^{3} V_k^L + \sum_{k=1}^{5} I_k + \sum_{k=1}^{5} I_k^D + \sum_{k=2}^{5} I_k^T + \sum_{k=2}^{5} I_k^F$$

Force of infection

$$\lambda = \frac{\rho}{N}\left(\sum_{k=1}^{5} R_k I_k + \sum_{k=1}^{5} R_k^V I_k^V + \sum_{k=1}^{5} R_k^D I_k^D + \sum_{k=2}^{5} R_k^T I_k^T + \sum_{k=2}^{5} R_k^F I_k^F\right)$$

$$\lambda_0^H = \lambda_0^L = \lambda$$
$$\lambda_k^H = (1-\phi_k^H)\lambda\,;k = 1,2,3$$
$$\lambda_k^L = (1-\phi_k^L)\lambda\,;k = 1,2,3$$
$$R_k = 1-(1-(1-\alpha_c)b_k)^{\frac{cn}{\rho}}(1-b_k)^{\frac{(1-c)n}{\rho}}$$
$$R_k^V = R_k^D = R_k^F = R_k$$
$$R_k^T = (1-\varepsilon_k)R_k$$

Vaccine efficacy profiles

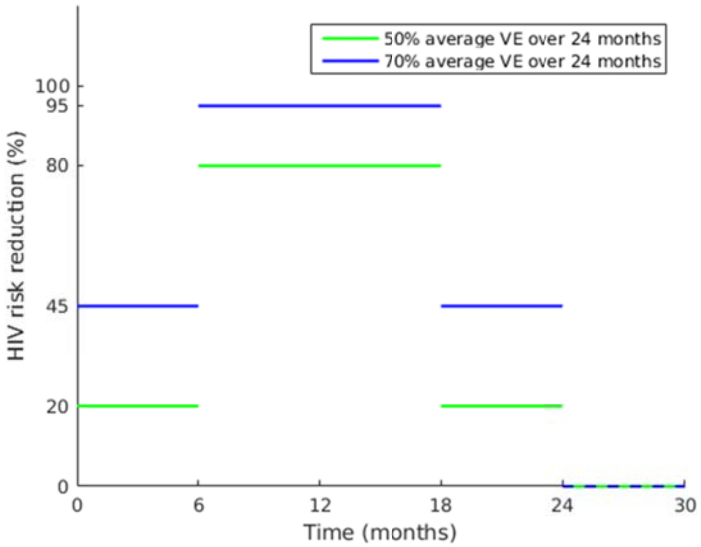

Figure S3 Efficacy profiles used in the analysis. Two vaccine efficacy profiles for the vaccine responders are simulated: one with 50% average VE over two years, assuming 20% HIV risk reduction over the first 6 months, 80% HIV risk reduction over the next 12 months and 20% HIV risk reduction over the last 6 months; another with 70% average VE over two years, assuming 45% HIV risk reduction over the first 6 months, 95% HIV risk reduction over the next 12 months and 45% HIV risk reduction over the last 6 months.

Assuming 72% responders and 28% non-responders in the susceptible population, we expect to observe a efficacy of 36% (= $50\% \cdot 72\% + 0\% \cdot 28\%$) with the first profile averaging 50% individual VE, and a efficacy of 50% (= $70\% \cdot 72\% + 0\% \cdot 28\%$) with the second profile averaging 70% individual VE in randomized controlled trials.

Note that the VE profiles are used to manage the flows between vaccinated compartments. With the flows based on the differential equations above, vaccinated individuals spend 24 months in average in vaccinated compartments and some of them remain protected after 24 months.

Model parameters

Parameters are color-coded. Green parameters are calibrated via random initialization and filtering. Yellow parameters are initial assumptions about VE. Uncolored parameters assume a fixed value which can be modified in secondary analysis.

Table S1. Parameter values and ranges used in the effectiveness analysis

Parameter	Description	Value	Range	Notes
$1/\nu_1$	Time in acute stage, HIV+ undiagnosed	0.21	± 0.05	Acute phase duration informed from (1)<br><br>Median durations from (2, 3) adjusted to obtain mean duration.<br><br>Uncertainty on total HIV/AIDS duration is distributed in each stage proportionally to mean duration.
$1/\nu_2$	Time in CD4>500, HIV+ undiagnosed	1.12	± 0.28	
$1/\nu_3$	Time in CD4 350-500, HIV+ undiagnosed	3.70	± 0.91	
$1/\nu_4$	Time in CD4 200-350, HIV+ undiagnosed	4.20	± 1.03	
$1/\nu_5$	Time in CD4<200, HIV+ undiagnosed	2.95	± 0.73	
ν_k^V	1/time in stage k, HIV+ vaccinated undiagnosed	ν_k		We assume vaccination has no impact on HIV progression speed.
ν_k^D	1/time in stage k, HIV+ diagnosed	ν_k		
ν_k^T	1/time in stage k, HIV+ on ART	$0.5\nu_k$		We assume that ART doubles the duration of HIV stages.
ν_k^F	1/time in stage k, HIV+ failed ART	ν_k		We assume that ART failure has no impact on HIV progression speed. (Here, failure also represents complete unlinking from care).
δ_k	Rate of diagnosis, HIV+ undiagnosed $k = 1,2,3,4$	Calibration	[0.1-0.15]	δ_k equal<br>Random uniform
δ_5	Rate of diagnosis, CD4<200 undiagnosed	Calibration	[0.4-0.6]	Random uniform<br>Faster diagnosis due to onset of AIDS
δ_k^V	Rate of diagnosis, HIV+ vaccinated	δ_k		
γ_5^D	Rate of ART initiation, CD4<200	Calibration	[0.5-1.5]	Random uniform
γ_4^D	Rate of ART initiation, CD4 [200-350]	Calibration	[0.5-1.5]	Random uniform<br>$\gamma_4^D < \gamma_5^D$, 2010-2015
γ_k^D	Rate of ART initiation, $k = 1,2,3$	Scenario dependent		= 0 during calibration

δ_5^T	Drop-out rate, CD4<200	Calibration	[0.01 – 0.1]	Random uniform
δ_k^T	Drop-out rate, CD4>200	Calibration	[0.01-0.1]	Random uniform ($\delta_4^T < \delta_5^T, \delta_2^T = \delta_3^T = \delta_4^T$) The rates of ART dropout are higher in the lowest CD4 categories to represents increased dropout for individuals having been on ART longer.
θ_5^T	Failure rate, CD4<200	Calibration	[0.01 – 0.1]	Random uniform
θ_k^T	Failure rate, CD4>200	Calibration	[0.01-0.1]	Random uniform ($\theta_4^T < \theta_5^T, \theta_2^T = \theta_3^T = \theta_4^T$)
π	Recruitment parameter (onset of sexual activity)	Calibration	[0.04-0.045]	$\pi = 0.04 + 0.005 \cdot M$ where M is the maximum of two uniform random numbers in the range [0, 1] Range informed from (4)
μ	Exit rate (natural death, aging)	Calibration	[0.02-0.025]	$\mu = 0.02 + 0.005 \cdot m$ where m is the minimum of the two uniform random numbers generated for π<br><br>Range informed from (4)
f_H	Fraction of population who will benefit from high vaccination efficacy	0.72		Vaccination scenario assumption.
τ_0^H	Vaccination rate in susceptible individuals who will have high vaccination efficacy	Scenario dependent.		Vaccination scenario assumption.
τ_0^L	Vaccination rate in susceptible individuals who will have low vaccination efficacy	Scenario dependent.		Vaccination scenario assumption.
$1/\tau_1^H$	Time in protection stage 1, high VE	0.5		Vaccination scenario assumption.
$1/\tau_2^H$	Time in protection stage 2, high VE	1		Vaccination scenario assumption.
$1/\tau_3^H$	Time in protection stage 3, high VE	0.5		Vaccination scenario assumption.

$1/\tau_1^L$	Time in protection stage 1, low VE	0.5		Vaccination scenario assumption.
$1/\tau_2^L$	Time in protection stage 2, low VE	1		Vaccination scenario assumption.
$1/\tau_3^L$	Time in protection stage 3, low VE	0.5		Vaccination scenario assumption.
ϕ_1^H	Relative protection conferred by vaccine, stage 1 high eff.	0.2		Vaccination scenario assumption.
ϕ_2^H	Relative protection conferred by vaccine, stage 2 high eff.	0.8		Vaccination scenario assumption.
ϕ_3^H	Relative protection conferred by vaccine, stage 3 low eff.	0.2		Vaccination scenario assumption.
ϕ_1^L	Relative protection conferred by vaccine, stage 1 low eff.	0		Vaccination scenario assumption.
ϕ_2^L	Relative protection conferred by vaccine, stage 2 low eff.	0		Vaccination scenario assumption.
ϕ_3^L	Relative protection conferred by vaccine, stage 3 low eff.	0		Vaccination scenario assumption.
ρ	Rate of partnership acquisition	Calibration	[1-1.5]	
n	Number of sex acts per year	Calibration	[95-120]	(5)
c	Proportion of condom use	0.2		(6)
α_c	Condom efficacy	0.7		(7)

b_1	Infection probability per act in HIV stage 1	0.0550		Values adjusted to obtain a distribution of infection flow by HIV stage comparable to other models in (8).<br><br>More details are given in the text below.
b_2	Infection probability per act in HIV stage 2	0.0021		
b_3	Infection probability per act in HIV stage 3	0.0006		
b_4	Infection probability per act in HIV stage 4	0.0011		
b_5	Infection probability per act in HIV stage 5	0.0033		
ε_k	Relative protection conferred by ART in HIV stage k	Calibration	[0.73-0.99]	ε_k equal<br>Random uniform<br>(9).

These coverage levels are implemented as follows. In the continuous vaccination scenario, we select a rate of vaccination such that, over two years, the desired coverage would be obtained in a cohort of constant size. In the campaign vaccination scenario, we vaccinate directly the corresponding fraction of the unvaccinated population. Note that, since some individuals are still protected by the vaccine after two years, the effective coverage is higher than the desired level just after a mass vaccination event (see Figure S4E).

The model is calibrated with 2012 epidemiologic data for South Africa (10) and informed by World Bank population data (4). Following a Monte-Carlo filtering scheme, we generate random parameter sets as described in Table S1, aiming to keep 1000 sets matching calibration data. We test parameter sets to ensure they respect an initial HIV incidence between 9 and 40 new infections per 1000 susceptible individuals in 2002 (10). Then, for each set, we run a simulation as follows.

1. The simulation's timeline starts in the year 2002.
2. The initial population size is 24.7 million.
3. The initial HIV prevalence is 15.6%.
4. We assume there was no ART available before 2002, such that initially no one is treated.
5. HIV+ individuals are distributed in HIV stages proportionally to stage mean duration.
6. HIV+ individuals with CD4<200 can initiate ART starting in 2002.
7. HIV+ individuals with CD4 [200-350] can initiate ART starting in 2010.
8. The model is simulated up to the beginning of 2015.

We select parameter sets with respect the following calibration targets (10).

1. The population size is within 3% of 28.2 million in 2012.
2. HIV prevalence is between 17.5% and 20.3% in 2012.
3. HIV incidence is between 13.8 and 20.6 per 1000 susceptible individuals in 2012.
4. The proportion of HIV+ individuals on ART (ART coverage) is between 25.6% and 32.5% in 2012.
5. The proportion of HIV+ individuals who are undiagnosed is between 45% and 55% in 2012.

The numbers for the infection probability per act by HIV stage have been set to obtain a distribution of infection flow by HIV stage similar to previous models in (8). Simulating our model with calibrated parameter sets but without any treatment ($\gamma_k^D = 0$), we have (in 2012) 16.2%-23.8% of transmission coming from HIV+ individuals with acute HIV, 13.6%-16.0% of transmission coming from HIV+ individuals with CD4>500, 11.3%-13.3% of transmission coming from HIV+ individuals with CD4 [350-500], 18.8%-21.2% of transmission coming from HIV+ individuals with CD4 [200-350], and 30.9%-34.8% of transmission coming from HIV+ individuals with CD4 < 200. This distribution is most similar to that of the EMOD and Portfolio models.

Epidemic scenarios used in the cost-effectiveness analysis

The cost-effectiveness analysis evaluates vaccination strategies under 3 epidemic scenarios:

1) The main epidemic scenario employs epidemic conditions based on the median incidence curve from the 1,000 simulated epidemics.

2) The optimistic epidemic scenario assumes higher ART coverage (52.3%) and almost perfect ART efficacy (97.4%) which implies that vast majority of the treated individuals are virally suppressed and results in more effective HIV prevention.

3) The pessimistic epidemic scenario assumes lower ART coverage (49.4%) and less effective ART (79% efficacy) which implies that large proportion of the treated individuals are virally unsuppressed and results in less effective HIV prevention.

3. Additional Results

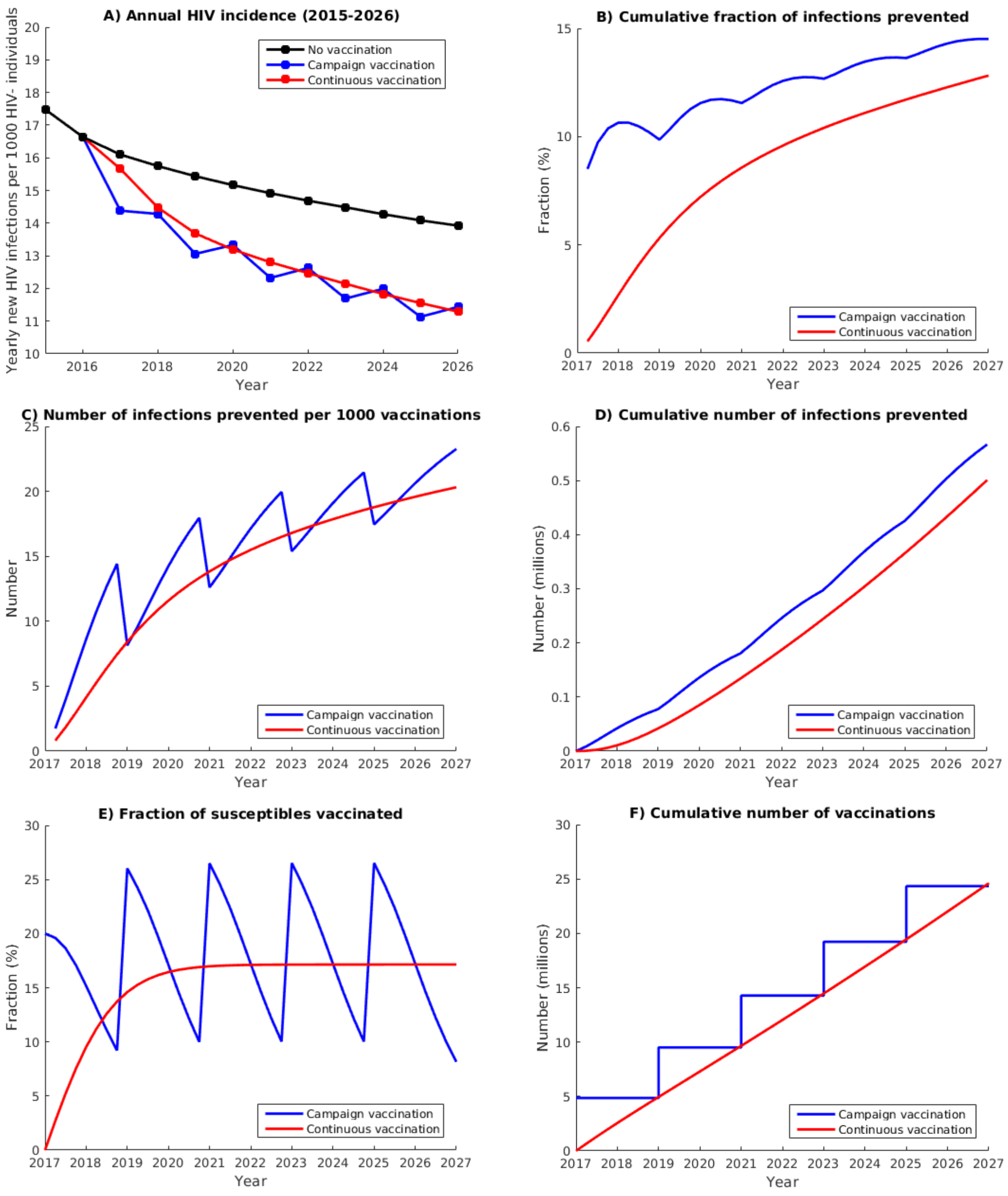

Figure S4. Temporal epidemic dynamics under scenarios without vaccination (black), with campaign vaccination (blue) and continuous vaccination (red). Curves represent.median values based on 1000 simulations in the main epidemic scenario with 70% vaccine efficacy and 20% vaccine coverage.

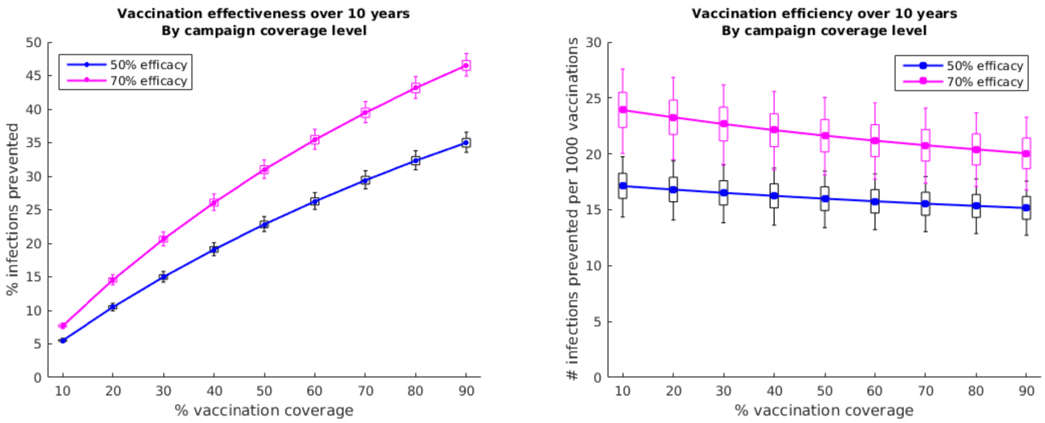

Figure S5. Effectiveness and efficiency of campaign vaccination for different levels of vaccination coverage.

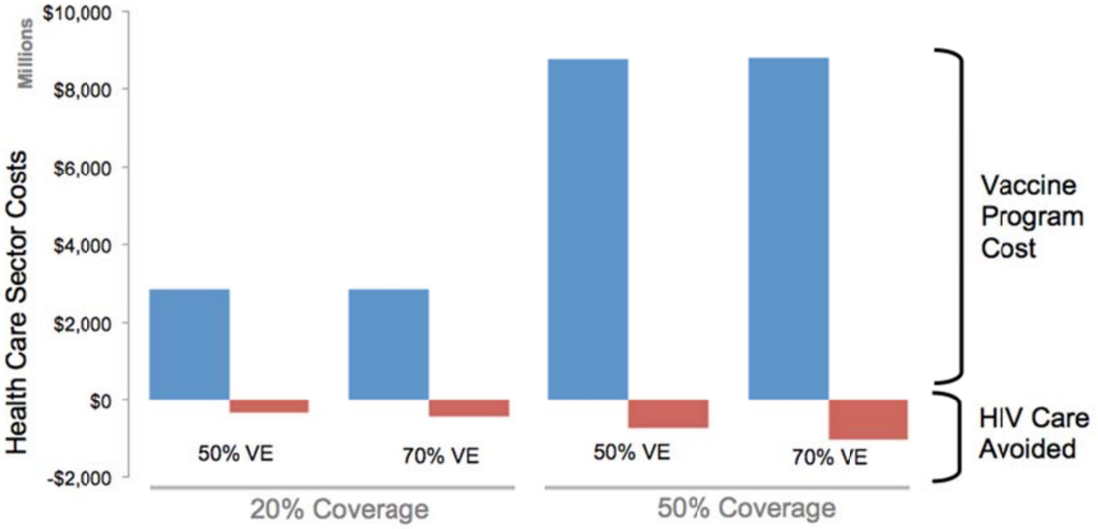

Figure S6. Health system costs disaggregated by the cost of vaccine delivery and HIV treatment, stratified by characteristics of the vaccine program, showing how the magnitude of vaccine program cost is larger than potential savings from HIV treatment avoided by reducing new infections.

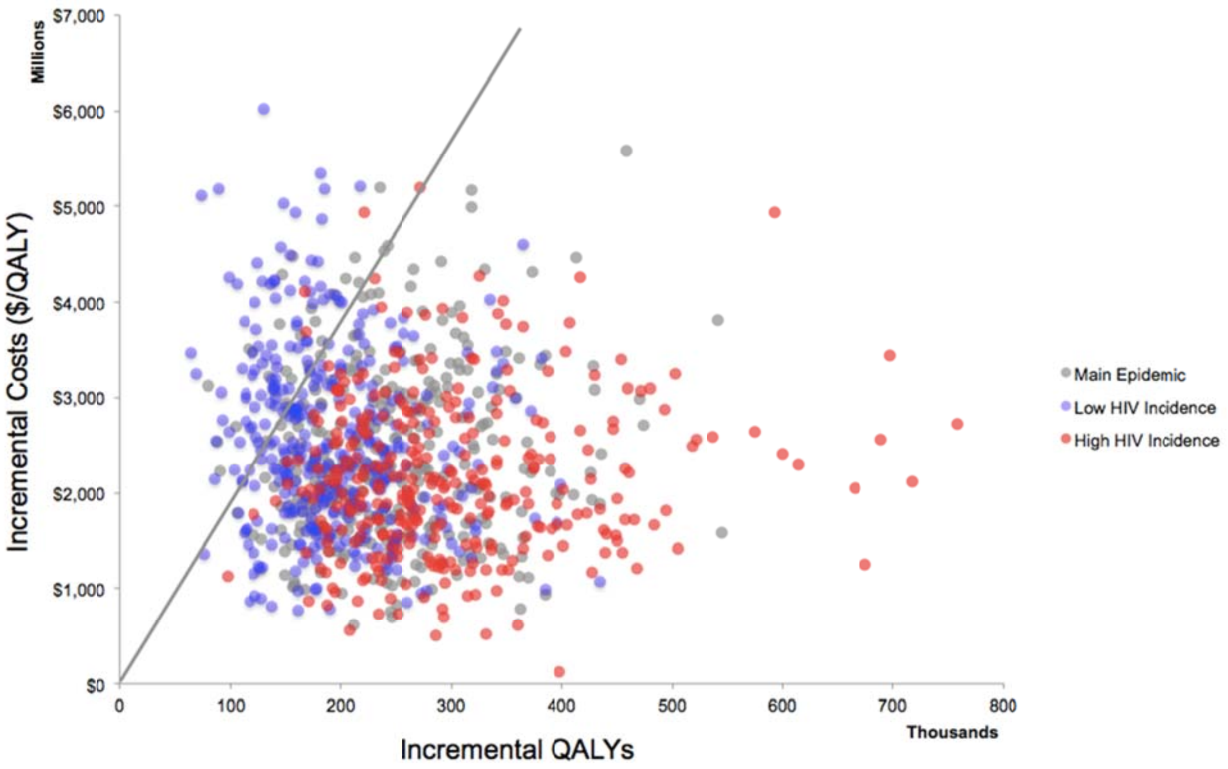

Figure S7. Probabilistic sensitivity analysis of the base-case scenario with 70% effective vaccine and 20% coverage for the main epidemic conditions (grey), optimistic epidemic conditions (blue), and pessimistic epidemic conditions (red). Second order Monte Carlo simulations use random draws for values based on selected and fitted distributions of cost, utility, and discount rate parameters. The grey line represents a willingness to pay threshold 3xGDP in South Africa.

Scenario analysis We further evaluate combined parameter uncertainty with different combinations of VE (50% and 70%), vaccination coverage (20% and 50%), implementation strategy (campaign and continuous), and epidemic settings (main, optimistic, and pessimistic). These dynamic transmission scenarios fed into a probabilistic sensitivity analysis of the economic evaluation. For each of the 24 dynamic transmission scenarios, 1,000 second order Monte Carlo simulations were conducted on cost, utility, and discount parameters. We selected and fit gamma distributions for costs and beta distributions for utilities and other parameters bounded by zero and one. Each simulation randomly draws a value based on the parameter distribution. ICERs presented in the manuscript reflect the mean of each stratum's simulations with 95% confidence ranges that are 1.96 times the standard error of the stratum's simulations. A cost-effectiveness acceptability curve was generated by varying the willingness-to-pay threshold and calculating the proportion of Monte Carlo simulations predicted to be cost-effective at each value.

Table S2. Comparison of the effectiveness and efficiency of the vaccination strategies from the main epidemic scenario with 70% VE and 20% coverage over 10 years and 30 years

Outcome metric	***over 10 years***	***over 30 years***
Cumulative fraction of infections prevented	Continuous : 12.8%<br>Campaign: 14.5%	Continuous : 20.8%<br>Campaign: 21.7%
Infections prevented per 1000 vaccinations	Continuous : 20.7<br>Campaign: 23.7	Continuous : 29.8<br>Campaign: 31.5

Table S3. Comparison of the effectiveness and efficiency over 10 years of the vaccination strategies from the main epidemic scenario with 70% VE and 20% coverage) to modified continuous strategy in which vaccination rate is increased during first 2 years.

Outcome metric	***Campaign main scenario***	***Continuous main scenario***	***Continuous +25% rate for 2y***	***Continuous +50% rate for 2y***
Cumulative fraction of infections prevented	14.5%	12.8%	13.6%	14.3%
Infections prevented per 1000 vaccinations	23.7	20.7	21.1	21.3

Table S4. Results from the deterministic scenario analyses ranging VE (50% and 70%), coverage (20% and 50%), vaccine price and epidemic settings.

		RANGE		Main epidemic scenario - $5 per Vaccine dose				Main epidemic scenario - $25 per Vaccine dose			
		Min	Max	50% VE and 20% Coverage		70% VE and 50% Coverage		50% VE and 20% Coverage		70% VE and 50% Coverage	
	**No Vaccine**			**Clinic-Based**	**Campaign**	**Clinic-Based**	**Campaign**	**Clinic-Based**	**Campaign**	**Clinic-Based**	**Campaign**
Vaccinated Adults (Millions)	-	23.54	59.40	24.68	24.40	59.40	56.13	24.68	24.40	59.40	56.13
Total Cost (Billions $)	$11.7	$13.7	$21.7	$13.7	$13.7	$16.5	$16.2	$15.9	$15.9	$21.7	$21.2
Total QALYs (Millions)	269.3	263.6	276.7	269.4	269.4	269.7	269.8	269.4	269.4	269.7	269.8
AIDS Deaths (Millions)	2.86	2.55	3.10	2.83	2.83	2.78	2.76	2.83	2.83	2.78	2.76
Incremental Cost (Billions $)	-	$2.0	$10.0	$2.0	$2.0	$4.9	$4.6	$4.2	$4.2	$10.0	$9.6
per person vaccinated ($)		$81.62	$172.40	$83.01	$83.40	$81.72	$81.62	$170.10	$172.40	$169.07	$170.80
per eligible adult ($)		$77.20	$380.89	$77.70	$77.20	$184.09	$173.76	$159.22	$159.57	$380.89	$363.63
Incremental QALYs, total	-	89,378	591,671	117,988	159,423	394,889	498,889	117,988	159,423	394,889	498,889
per person vaccinated		0.0035	0.0109	0.0048	0.0065	0.0066	0.0089	0.0048	0.0065	0.0066	0.0089
per eligible adult		0.0032	0.0234	0.0045	0.0060	0.0150	0.0189	0.0045	0.0060	0.0150	0.0189
AIDS Deaths Avoided	-	17,449	118,424	23,001	31,678	77,968	99,797	23,001	31,678	77,968	99,797
ICER ($/QALY)		$9,183	$36,882	$17,364	$12,767	$12,291	$9,183	$35,580	$26,390	$25,431	$19,217

Table S4 continued

		Optimistic epidemic scenario				Pessimistic epidemic scenario			
		50% VE and 20% Coverage		70% VE and 50% Coverage		50% VE and 20% Coverage		70% VE and 50% Coverage	
	**No Vaccine**	**Clinic-Based**	**Campaign**	**Clinic-Based**	**Campaign**	**Clinic-Based**	**Campaign**	**Clinic-Based**	**Campaign**
Vaccinated Adults (Millions)	-	25.88	25.49	25.88	25.49	23.72	23.54	57.25	54.27
Total Cost (Billions $)	$11.7	$14.9	$14.9	$14.9	$14.9	$15.6	$15.6	$19.7	$19.3
Total QALYs (Millions)	269.3	276.6	276.7	276.6	276.7	263.6	263.6	263.9	264.0
AIDS Deaths (Millions)	2.86	2.56	2.55	2.56	2.55	3.10	3.09	3.04	3.01
Incremental Cost (Billions $)	-	$3.3	$3.3	$3.3	$3.3	$3.0	$3.0	$7.1	$6.7
per person vaccinated ($)		$127.35	$128.98	$127.35	$128.98	$125.09	$125.95	$123.32	$123.55
per eligible adult ($)		$118.95	$118.62	$118.95	$118.62	$117.26	$117.18	$279.03	$265.01
Incremental QALYs, total	-	89,378	122,588	89,378	122,588	141,302	189,275	472,264	591,671
per person vaccinated		0.0035	0.0048	0.0035	0.0048	0.0060	0.0080	0.0082	0.0109
per eligible adult		0.0032	0.0044	0.0032	0.0044	0.0056	0.0075	0.0187	0.0234
AIDS Deaths Avoided	-	17,449	24,356	17,449	24,356	27,519	37,645	93,121	118,424
ICER ($/QALY)		$36,882	$26,817	$36,882	$26,817	$20,997	$15,665	$14,949	$11,333

4. Glossary of technical terms, health metrics and abbreviations

annual HIV incidence: ratio of the number of yearly new HIV infections to mid-year number of HIV negative individuals

cost-effectiveness analysis: An analytic tool in which the costs and effects of a program and at least 1 alternative are calculated and presented in a ratio of incremental cost to incremental effect. Effects are health outcomes, such as cases of a disease prevented, years of life gained, or quality-adjusted life-years, rather than monetary measures as in cost-benefit analysis.

cost-effectiveness threshold: cost-effective interventions are defined as meeting a threshold per QALY averted of 1-3 times the annual GDP per capita, or the maximum ICER at which the given intervention is considered cost-effective given the payer willingness to pay for health.

discounting: The process of converting future dollars and future health outcomes to their present values.

health care payer perspective: A viewpoint for conducting a cost-effectiveness analysis that includes formal health care sector (medical) costs borne by payers. These medical costs include current and future costs related to the condition under consideration. It does not include paid out-of-pocket costs to patients or costs unrelated to HIV.

health utility: A representation of strength of preference for a given health-related outcome on a cardinal numeric scale, where a value of 1.0 represents full health, 0.0 represents dead, and negative values represent states worse than dead

HIV prevalence: ratio of the number of HIV positive individuals to the total number of individuals in the population at a given time.

incremental cost-effectiveness ratio (ICER): The ratio of the difference in costs between 2 alternatives to the difference in effectiveness between the same 2 alternatives calculated as

$$\mathrm{ICER} = \frac{\mathrm{Cost}_{\mathrm{intervention}} - \mathrm{Cost}_{\mathrm{standard\ of\ care}}}{\mathrm{QALY}_{\mathrm{intervention}} - \mathrm{QALY}_{\mathrm{standard\ of\ care}}}$$

net monetary benefit (NMB): Linear combination of costs and effects, expressed in US dollars. NMB of a vaccine campaign compared to continuous vaccination is defined by:

$$\mathrm{NMB} = (\mathrm{QALYs}_{\mathrm{campaign}} - \mathrm{QALYs}_{\mathrm{continuous}}) * \mathrm{WTP} - (\mathrm{Cost}_{\mathrm{campaign}} - \mathrm{Cost}_{\mathrm{continuous}})$$

positive externality: a benefit that is enjoyed by a third-party as a result of an economic transaction, such as unvaccinated individuals being a third party indirectly affected by the vaccination of others.

quality-adjusted life years (QALYs): a generic measure of disease burden, including both the quality and the quantity of life lived. It is used in economic evaluation to assess the value for money of medical interventions. One QALY equates to one year in perfect health.

time horizon: the duration of future time at which costs and benefits are considered.

uncertainty interval (90% UI): The range comprised between the 5th percentile and the 95th percentile of the values for a outcome metric obtained with the model using 1000 calibrated parameter sets .

vaccine efficacy (VE): reduction of HIV susceptibility as a result of the vaccination. We simulate vaccine efficacy profiles for the vaccine responders which account for the schedules of the multi-dose regimens (Supplementary Figure S3).

vaccine effectiveness: fraction of cumulative new HIV infections prevented over a specified period calculated as

$$1 - \frac{\text{Cumulative number of new infections (with vaccination)}}{\text{Cumulative number of infections (no vaccination)}}$$

vaccine efficiency: number of cumulative new HIV infections prevented per 1000 cumulative vaccinations over a specified period calculated as

$$1000 * \frac{\text{Cumulative number of new infections (with vaccination)}}{\text{Cumulative number of vaccinations}}$$

willingness-to-pay (WTP): the maximum monetary value a payer is willing to pay for a unit of health benefit.

Several definitions are adapted from the Recommendations from the Second Panel on Cost-Effectiveness in Health and Medicine (11).

References

1. Wawer MJ*, et al.* (2005) Rates of HIV-1 transmission per coital act, by stage of HIV-1 infection, in Rakai, Uganda. *Journal of Infectious Diseases* 191(9):1403-1409.
2. Todd J*, et al.* (2007) Time from HIV seroconversion to death: a collaborative analysis of eight studies in six low and middle-income countries before highly active antiretroviral therapy. *AIDS* 21:S55-S63.
3. Lodi S, et al. (2011) Time from Human Immunodeficiency Virus seroconversion to reaching CD4+ cell count thresholds < 200, < 350, and < 500 cells/mm^3 : Assessment of need following changes in treatment guidelines. *Clinical Infectious Diseases* 53:817–825.
4. The World Bank. Health Nutrition and Population Statistics (2015) Female population 15-19, 20-24, 25-29, 30-34, 35-39, 40-44, 45-49 and Male population 15-19, 20-24, 25-29, 30-34, 35-39, 40-44, 45-49 in South Africa 1970-2014.
5. Smith J*, et al.* (2014) The Distribution of Sex Acts and Condom Use within Partnerships in a Rural Sub-Saharan African Population. *PLOS ONE* 9(2):e88378-.
6. Beksinska, M. E., Smit, J. A. & Mantell, J. E. Progress and challenges to male and female condom use in South Africa. *Sexual Health* 9, 51-58 (2012).
7. Davis, K. R. & Weller, S. C. The Effectiveness of Condoms in Reducing Heterosexual Transmission of HIV. *Family Planning Perspectives* 31, 272-279 (1999).
8. Eaton JW*, et al.* (2012) HIV Treatment as Prevention: Systematic Comparison of Mathematical Models of the Potential Impact of Antiretroviral Therapy on HIV Incidence in South Africa. *PLoS Med* 9(7):e1001245-.
9. Cohen MS*, et al.* (2011) Prevention of HIV-1 Infection with Early Antiretroviral Therapy. *New England Journal Of Medicine* 365(6):493-505.
10. O.Shisana TR, L.C.Simbayi, K.Zuma, S.Jooste, N.Zungu, D.Labadarios, D.Onoya (2014) *South African National HIV Prevalence, Incidence and Behaviour Survey, 2012* (HSRC Press, Cape Town, South Africa).
11. Sanders GD, Neumann PJ, Basu A, Brock DW, Feeny D, Krahn M, Kuntz KM, Meltzer DO, Owens DK, Prosser LA, Salomon JA, Sculpher MJ, Trikalinos TA, Russell LB, Siegel JE, Ganiats TG. Recommendations for Conduct, Methodological Practices, and Reporting of Cost-effectiveness Analyses. Second Panel on Cost-Effectiveness in Health and Medicine. *JAMA*. 2016;316(10):1093–1103. doi:10.1001/jama.2016.12195
